# Editorial: Phenotypic and functional heterogeneity of neutrophils

**DOI:** 10.3389/fimmu.2023.1195605

**Published:** 2023-04-04

**Authors:** Nicola Tamassia, Sebastien Jaillon

**Affiliations:** ^1^ Section of General Pathology, Department of Medicine, University of Verona, Verona, Italy; ^2^ Department of Biomedical Sciences, Humanitas University, Pieve Emanuele, Milan, Italy; ^3^ IRCCS Humanitas Research Hospital, Rozzano, Milan, Italy

**Keywords:** neutrophils, innate immunity, inflammation, cancer, granulocytes

Neutrophils represent the most abundant immune cells in human circulation and are rapidly recruited to sites of inflammation and infection where, exerting effector functions such as degranulation, production of reactive oxygen species, phagocytosis, and formation of neutrophil extracellular traps, they represent the first line of host defense. In addition, neutrophils can also participate in the orchestration of the inflammatory and immune response by the release of inflammatory mediators, such as cytokines, chemokines, prostaglandins and leukotrienes, as well as their interaction with other leukocytes of both the innate and adaptive arms of the immune response (1, 2). For a long time, neutrophils have been considered to be a homogeneous population at the phenotypical and functional level. However, recent advances obtained by the use of novel technologies, such as multiparametric flow cytometry and more recently scRNAseq, have uncovered an unexpected heterogeneity of neutrophils in both pathological and physiological conditions (3).

In this Research Topic, five articles provide interesting new insights regarding the heterogeneity of neutrophils in different pathological conditions, including tumors, cystic fibrosis, psoriasis and *in vitro* models of infection.

Neutrophil heterogeneity has been particularly studied in tumors where different subsets have been identified, including neutrophils with pro-tumor and anti-tumor activity (3). With these premises, neutrophil subpopulations may be important candidates for the identification of novel cellular and molecular biomarkers in tumors. In this context, the study by Cristinziano et al. evaluated the expression of programmed death-1 (PD) Ligand 1 (PD-L1) in neutrophils from stage IV melanoma patients, which have excellent benefits from treatment with monoclonal antibodies targeting the PD-1/PD-L1 axis. Neutrophils from melanoma patients displayed an activated phenotype associated with increased expression of PD-L1 compared to healthy donors. Importantly, in patients undergoing immunotherapy, a lower frequency of PD-L1^+^ neutrophil was associated with better outcomes compared to patients with a higher frequency of PD-L1^+^ neutrophils. In addition, stratification of melanoma patients based on the presence or absence of mutations in the oncogene *BRAF*, revealed that high PD-L1^+^ neutrophil frequency was correlated with poor progression-free survival and poor overall survival only in *BRAF* wild-type patients. The identification of factors that may effectively predict the immunotherapy response of cancer patients, including melanoma patients, has become a priority in cancer treatment and the study by Cristinziano et al. provided an interesting candidate.

An increased proportion of neutrophils expressing high levels of PD-L1, together with CD114, the receptor for G-CSF, was also observed in the blood of cystic fibrosis patients. In this Research Topic, this subset was identified by Martin et al. using an unsupervised analysis of flow cytometry data obtained from the analysis of a large panel of membrane markers. Interestingly, this study showed that in cystic fibrosis patients, during pulmonary exacerbation, an expansion of low density neutrophils associated with an increase in the frequency of normal density CD16^low^/CD62L^high^ neutrophils could be observed. This neutrophil heterogeneity might affect the disease course or clinical outcome, and neutrophils subsets could constitute therapeutic targets by increasing their antibacterial potential and limiting their immune-suppressive capacities.

In this collection, Costa et al. identified a possible involvement of neutrophils in a mouse model of psoriasiform dermatitis. Indeed, increased epidermal thickening was observed in neutrophil-depleted mice. Importantly, this phenotype was associated with alteration of the inflammatory response and increased presence of IL-17-producing γδ T cells. Further experiments showed that neutrophils can inhibit the production of IL-17 by γδ T cells through a cell-contact dependent production of reactive oxygen species. Neutrophil activation and production of reactive oxygen species was dependent on intracellular signaling transmitted by a member of nonreceptor tyrosine kinase referred to as spleen tyrosine kinase. Therefore, this study uncovers a novel possible role for neutrophils in controlling γδ T cells activation during psoriasis and suggests that targeting neutrophils may be a potential therapeutic strategy for psoriasis.

The notion of neutrophil plasticity derives from their ability to respond to a variety of exogenous and endogenous mediators by modifying their transcriptomic profile, phenotype and effector functions. Some pathogens exploit neutrophil plasticity as part of their virulence strategy. For instance, *Helicobacter pylori* can induce differentiation of neutrophils characterized by a profound nuclear hypersegmentation and the secretion of proinflammatory cytokines (4). In this Research Topic, Prichard et al. used sophisticated imaging methods to show that *Helicobacter pylori*-infected neutrophils exhibited impaired chemotaxis and uropod retraction. In contrast, chemotactic receptor abundance, adhesion and phagocytosis were unchanged. Importantly, impaired migration of *Helicobacter pylori*-infected cells was attributable to a defect in dynamic changes of myosin IIA contractility. These results could explain why *Helicobacter pylori* infections persist, as the impairment of neutrophil activity may impede the protective response against the bacteria.

Another interesting paper in this collection describes new insights about a recently identified subset of activated neutrophils expressing the dual endothelin-1/signal peptide receptor (DEspR). This subset was found more abundant in patients with acute respiratory distress syndrome and was significantly associated with disease severity and patient mortality (5). DEspR^+^CD11b^+^CD66b^+^ neutrophils displayed reduced spontaneous apoptosis, which can be associated with delayed efferocytosis and resolution of inflammation. In this collection, Carstensen et al. assessed the presence of DEspR^+^CD11b^+^CD66b^+^ neutrophils, defined as a “rogue” subset, in models of lipopolysaccharide (LPS)-induced inflammation in healthy human volunteers, macaques and rats. Increased DEspR^+^CD11b^+^CD66b^+^ neutrophil frequency was observed in bronchoalveolar lavage fluid in healthy volunteers after segmental LPS-challenge. A treatment with anti-DEspR antibodies reduced DEspR^+^CD11b^+^CD66b^+^ neutrophil count associated with increased survival in the model of LPS-encephalopathy in rat and reduced hypoxemia in LPS-induced transient acute lung injury in macaques. Therefore, the study by Carstensen et al. provided evidence on the potential clinical benefit of antibody-based therapy against DEspR for the treatment of neutrophil-mediated acute tissue injury.

In conclusion, the studies included in this Research Topic highlight that neutrophil subsets can represent useful biomarkers for diagnosis and prognosis of different immune-related diseases. In addition, the functional diversity of neutrophil subsets offers the possibility of selective therapeutic intervention which may be used in the treatment of various neutrophil-driven diseases.

## Author contributions

All authors listed have made a substantial, direct, and intellectual contribution to the work and approved it for publication.
